# Resource impact of bronchiectasis and associated exacerbations: based on a 5-year prospective observational cohort study (BronchUK)

**DOI:** 10.1136/bmjresp-2026-004203

**Published:** 2026-07-14

**Authors:** Carlos Gallego-Moll, Laia Maynou, Phil Mawson, Martin Kelly, Joseph Stuart Elborn, Adam T Hill, Timothy Gatheral, Anita Sullivan, Charles Haworth, John R Hurst, Jeremy Stuart Brown, Mary P Carroll, Michael R Loebinger, Judy M Bradley, Paul Walker, John Steer, Jamie Duckers, James D Chalmers, Richard McNally, Anthony De Soyza, Alistair McGuire

**Affiliations:** 1 Department of Econometrics, Statistics and Applied Economics, University of Barcelona, Barcelona, Spain; 2 Public University of Navarre Institute for Advanced Research in Business and Economics, Pamplona, Spain; 3 Center for Research in Health and Economics, Universitat Pompeu Fabra, Barcelona, Spain; 4 Department of Health Policy, The London School of Economics and Political Science, London, UK; 5 Newcastle University, Newcastle upon Tyne, UK; 6 Altnagelvin Hospital, HSC Western Health and Social Care Trust, Londonderry, UK; 7 Queen’s University Belfast, Belfast, UK; 8 University of Edinburgh MRC Centre for Inflammation Research, Edinburgh, UK; 9 Respiratory Medicine, University Hospitals of Morecambe Bay NHS Foundation Trust, Kendal, UK; 10 University Hospitals Birmingham NHS Foundation Trust, Birmingham, UK; 11 Papworth Hospital NHS Foundation Trust, Cambridge, UK; 12 Department of Respiratory Medicine, UCL, London, UK; 13 University Hospital Southampton NHS Foundation Trust, Southampton, UK; 14 University of Southampton, Southampton, UK; 15 Royal Brompton Hospital, London, UK; 16 Imperial College London National Heart and Lung Institute, London, UK; 17 University of Liverpool, Liverpool, UK; 18 Northumbria Healthcare NHS Foundation Trust, Tyne and Wear, UK; 19 University Hospital Llandough, Llandough, UK; 20 University of Dundee, Dundee, UK

**Keywords:** Bronchiectasis, Health Economist

## Abstract

**Background:**

Our objective is to document major healthcare resource use and associated costs in the care of patients with bronchiectasis, with a particular focus on the costs incurred by exacerbations.

**Methods:**

We use a unique dataset from the Bronchiectasis Observational Cohort and Biobank UK. The study includes a baseline cohort of 1119 patients with a primary diagnosis of bronchiectasis, followed for up to five years. Data were extracted and linked to centrally held National Health Service (NHS) resource use data.

**Results:**

The average age of the cohort was 64 years and 62% were female. The most common bronchiectasis aetiology was idiopathic or post-infectious. Across follow-ups, exacerbations became less frequent: the proportion of patients with no events increased for all settings, while recurrent (≥2) events declined markedly and single-event categories remained largely stable. Exacerbations were predominantly managed in primary care settings. Based on exacerbation frequency and type and applying 2024/2025 NHS reference costs, the estimated total healthcare exacerbation costs for the study population ranged from £2 million to £3.2 million. Disaggregated estimates for the highest cost scenario include: general practitioner (GP)-only exacerbations (n=2824 events; £479 945), emergency department-managed exacerbations (n=311; £260 126) and inpatient-managed exacerbations (n=680; £2 428 291). The average estimated cost per patient over the full period was £1943. This corresponds to a weighted annual average of £495 per patient (including patients with no exacerbations), with most costs attributable to inpatient-managed exacerbations. Specifically, the average cost was £2980 for individuals with one or more exacerbations, £4865 for those with two or more and £5875 for those with three or more.

**Conclusions:**

The findings highlight the economic burden of bronchiectasis exacerbations and demonstrate that targeted management strategies to reduce exacerbation rates should increase health benefits and substantially reduce healthcare costs associated with bronchiectasis.

**WHAT IS ALREADY KNOWN ON THIS TOPIC**

- Bronchiectasis is a prevalent chronic respiratory condition with a growing global burden, yet an underreported illness and health economic data are largely derived from non-UK healthcare systems. Exacerbations are a critical feature of the disease, not only because their symptoms can be prolonged and debilitating but also because they independently predict future mortality and drive substantial health service usage.

**WHAT THIS STUDY ADDS**

- While exacerbations are known to drive disease progression, their specific impact on healthcare resource utilisation and costs within health services remains poorly quantified. This prospective cohort study establishes that inpatient hospitalisations for exacerbations account for nearly 80% of direct healthcare costs in the UK. We identify a distinct cost-escalation gradient, where annual per-patient expenditure rises more than 10-fold—from a weighted average of £495 to over £5800—in patients experiencing three or more severe episodes annually.

**HOW THIS STUDY MIGHT AFFECT RESEARCH, PRACTICE OR POLICY**

- These findings provide the first robust UK reference costs for bronchiectasis, serving as an essential baseline for cost-effectiveness analyses of emerging therapies. The data underscore that clinical management strategies specifically targeting the reduction of exacerbation frequency and hospitalisations offer the greatest potential to alleviate the substantial economic burden on the NHS.

## Introduction

 Non-cystic fibrosis (CF)-related bronchiectasis (BE) is a chronic respiratory disease that is characterised by bronchial dilation with symptoms of a chronic cough, sputum production and, commonly, acute infections called ‘exacerbations’. Global prevalence appears to be increasing, though limited access to confirmatory CT scanning and diagnostic overlap with Chronic obstructive pulmonary disease (COPD) or asthma likely led to under-reporting.^1^

Global estimates of the prevalence of BE, excluding people with CF, give widely divergent estimates across countries due to underlying heterogeneity in the disease aetiology and access to imaging for appropriate diagnosis. The association of BE with socioeconomic deprivation may also lead to under-presentation of affected individuals.^2^ Two UK studies using general practitioner databases reported increasing prevalence, reaching 379–566 per 100 000 for women and 281–485 per 100 000 for men by 2012–2013.^3 4^ The latter study also records age-adjusted mortality rates that are more than twice as high for BE patients compared with the general UK population.

Data on the burden of disease, including healthcare resource utilisation and associated costs for the UK are scant in BE, with most studies originating from the USA,^5^ Germany and Australia.^6^ A systematic review indicates substantial medical costs mostly linked to hospitalisations, treatments and emergency department (ED) and outpatient visits. Indirect costs included sick pay and lost income and/or productivity.^7^ Exacerbations are a critical feature of the disease, not only because their symptoms can be prolonged and debilitating, but also because they independently predict future mortality and drive substantial health service usage.^7^

Against the background of limited research on the prevalence and treatment of BE the Bronchiectasis Observational Cohort and Biobank UK (BronchUK) study was initiated.^8^ This is a multicentre, prospective, cohort study of patients with a clinical history consistent with symptomatic BE and a confirmatory CT scan. Patients were identified and recruited from fifteen secondary care outpatient clinics in either general respiratory or specialised hospital clinics across the UK with a follow-up period of 5 years (starting in 2020). Our primary objective is to document major resource usage over the study period, specifically focusing on the role of economic burden of exacerbations.

## Methods

BronchUK has baseline data and long-term annual follow-up over a minimum of a 5-year period with a range of objectives including the description of treatment patterns and healthcare utilisation across the UK. It is run in conjunction with and parallel to the European Multicentre Bronchiectasis Audit and Research Collaboration (EMBARC) project that has created a complementary set of BE registries, and one of the exclusion criteria for BronchUK was for patients already enrolled in EMBARC. The other exclusion criteria were patients with CF or where BE was not the main or co-dominant respiratory disease or those with prior lung transplantation due to BE were excluded.

In line with the observational status of the study, the aetiology of BE is based on diagnosis by the physician caring for the patient. The data are based on a patient identifier that allows linkage to NHS and ONS data and is held within a ‘Safe Haven’ at the Health Informatics Centre based at the Farr Institute, University of Dundee to ensure patient anonymity is maintained. After the collection of baseline data, subsequent data on patients were collected on an annual basis (within a 6-month variance) as aligned to routine clinical visits for a 5 years follow-up, unless the patient is discharged or lost due to withdrawal.

A wide range of data on the aetiological testing performed for diagnosis, microbiology testing, severity of disease, comorbidities, lung function, radiology, treatments, exacerbations, quality of life and patient characteristics (including smoking status) were collected.^9^ The severity of disease data included the variables required to calculate the Bronchiectasis Severity Score, such as history of hospitalisations. Treatment data recorded oral and inhaled therapies and maintenance antibiotics (systemic or inhaled), mucoactive drugs and anti-inflammatories. Comorbidities are collected within the database and we analysed comorbidity counts (CC). Physiotherapy and vaccination status were also recorded. Radiological data included historical CT scans recorded at baseline, as well as subsequent scans.^9^

We analysed the association between exacerbations and healthcare resource usage and followed the EMBARC pragmatic definition of an exacerbation which includes self-managed exacerbation where antibiotics are used after sustained increase in symptoms (primary care managed). Severe exacerbations are defined in line with the BTS criteria as an exacerbation requiring hospital admission. Emergency room visits are also recorded. At baseline and at each scheduled follow-up visit, participants reported whether they had experienced any BE exacerbation in the preceding 12 months, as exact dates of exacerbation events were not collected. Variables with >30% missing data were excluded. We used a parsimony criterion for this exclusion, as we could not distinguish whether the missing data arose from a lack of information, a lack of changes or if the data were missing at random. No imputation was performed and missing values were retained, except for exacerbation outcomes, where records with missing data were removed.

The BronchUK data were supplemented with data on treatments from the National Institute for Health and Care Excellence (NICE) and treatment costs from NHS and Personal Social Services Research Unit (PSSRU) cost sources. The emergency room and in-patient visits were based on costs taken from the 2023/2025 NHS Payment Scheme (2024/2025 prices workbook) which is a detailed costing of all NHS treatments based on the tariff prices paid to hospitals by primary care commissioning groups. All documentation is found at the NHS England website^10^ and those costs used are detailed below.

The cost of primary care is drawn from the Unit Costs of Health and Social Care 2023 Manual as downloaded from the Kent University Repository.^11^ All costs are in 2024/2025 prices, with the primary care costs, as they are given in 2022/2023 prices, uplifted by an inflation factor of 6.1 as given in the NHS Digital Financial Performance Update, 2024.^12^

### Study outcomes and statistical analysis

The primary outcomes analysed in this study were resource utilisation by exacerbation type and the occurrence of any subsequent exacerbation during follow-up. We summarised the baseline characteristics of the patients, stratifying them according to the most severe exacerbation experienced in the year prior to the study. This stratification was selected to map a distinct hierarchy of healthcare resource intensity and the associated accumulation of costs required for the NICE cost-effectiveness framework. By categorising events from no exacerbation (0) to primary care management (1) and acute hospital intervention (2/3), the model accurately captures the discrete escalation in clinical severity and economic burden characteristic of the BE frequent-exacerbator phenotype. Participants were classified into four mutually exclusive groups, with each patient assigned to the highest applicable category of healthcare utilisation: (0) no exacerbations, (1) exacerbations with no hospitalisation/ED, (2) exacerbations with ED and (3) exacerbations with hospitalisation. Categorical variables were assessed using χ² or Fisher’s exact tests as appropriate. Continuous variables were compared using analysis of variance when normality assumptions were met, and Kruskal-Wallis tests otherwise.

We also focused on the longitudinal distribution of the frequency and severity of exacerbations throughout the follow-up period. For each interval between study visits, the number of exacerbations per participant was tallied, and the frequency was classified as zero, one or two or more events. Additionally, these events were stratified by severity based on the level of healthcare required, differentiating between those managed in primary care or self-managed, those requiring an ED visit without admission and those that led to hospitalisation. This longitudinal approach allowed for the characterisation of temporal changes in both the frequency and severity of exacerbations over time.

Given unbalanced follow-ups, our approach was to run statistical regressions to determine association between the baseline exacerbation type (ie, history in the previous year) and exacerbations during the follow-up period.

Logistic regression models run for each exacerbation type are adjusted for patient characteristics:


$$Y_{i}=\alpha +\beta_{1}Exacer_{i}+\beta_{2}ExacerwithED_{i}+\beta_{3}Exacerwithhosp_{i}+\delta X_{i}+\epsilon_{i}$$

Resultant odds of experiencing an exacerbation during follow-up (binary outcome), stratified by baseline exacerbation history of each respective type were estimated and are presented below. Patient characteristics included sex, ethnicity, age, smoking status, cardiovascular disease, history of atypical mycobacterial infection, pneumonia and modified Medical Research Council score. To account for varying follow-up lengths, the number of observed visits was also included as a covariate. Regression results are provided in online supplemental material 1.

Finally, we present cost estimates related to the exacerbation status and exacerbation type based on data on treatments from NICE and treatment costs from NHS and PSSRU cost sources. In determining the costs of treatment, we note that the study data are derived from a multicentre sample of UK BE patient population seen in secondary care centres and not a representative sample of those seen in all hospital or primary care settings. In what follows we adopt extremely conservative treatment estimates as associated with this sample population. This reflects the small numbers observed when recording more than two exacerbations suffered by an individual. Due to anonymity restrictions this also meant censoring the data when individuals suffered two or more exacerbation episodes within a year.

In estimating treatment costs conservatively, we have assumed that those suffering at least three exacerbation episodes suffered no more than this number, although we know that there will be a long tail in the distribution of individuals suffering exacerbations and therefore a number of much higher sufferers, within this patient population. While a conservative approach, this simplifies the analysis by being consistent with our previous classification. We treated registry data as cross-sectional, assuming homogeneity of treatment costs within each severity level. Inpatient costs were weighted by complication (elective or emergency admission) and co-morbidity (CC) scores to reflect clinical severity.

## Results

Follow-up participation declined from 1119 (baseline) to 252 (fifth follow-up). Missing data were <10% for all variables except daily sputum volume (29%). To understand the structure of our observation periods, the mean follow-up duration was 4.49 visits (SD 1.47), with a range of 1 to 6 visits (years). Table 1 summarises clinical characteristics at baseline. We stratified the cohort into four mutually exclusive groups, each patient assigned to the highest applicable category of healthcare utilisation: 0 indicating no exacerbations across the totality of their follow-up; 1 indicating exacerbations not requiring hospitalisation through the ED or in-patient; 2 exacerbations requiring ED visits but not inpatient hospitalisation and 3 indicating exacerbations requiring hospitalisation. Patients with no exacerbations were the oldest (mean ≈67 years) yet reported slightly shorter disease duration, whereas the ED-only group was the youngest (≈61 years).

**Table 1:** Baseline characteristics across patient groups

Patients group	0–no exacerbations (N=79)	1–exacerbations with no hosp/ED (N=564)	2–exacerbations with ED (N=79)	3–exacerbations with hospitalisation (N=397)	Total cohort (N=1119)	P value
Gender						0.258
Female	43 (54.4%)	364 (64.5%)	51 (64.6%)	240 (60.5%)	698 (62.4%)	
Ethnicity						**0.001**
White	69 (87.3%)	531 (94.1%)	73 (92.4%)	387 (97.5%)	1060 (94.7%)	
Age at consent						**0.015**
Mean (SD) (range)	67.4 (13.3) (19.0–87.0)	64.2 (12.3) (18.0–86.0)	60.9 (15.7) (18.0–85.0)	64.7 (12.9) (18.0–88.0)	64.4 (12.9) (18.0–88.0)	
How long has the patient had bronchiectasis (years)						**0.015**
<5	32 (40.5%)	199 (35.3%)	29 (36.7%)	113 (28.5%)	373 (33.3%)	
5–10	18 (22.8%)	112 (19.9%)	14 (17.7%)	65 (16.4%)	209 (18.7%)	
10–15	9 (11.4%)	68 (12.1%)	8 (10.1%)	48 (12.1%)	133 (11.9%)	
15–20	<5 (5.1%)	38 (6.7%)	8 (10.1%)	36 (9.1%)	86 (7.7%)	
>20	9 (11.4%)	106 (18.8%)	19 (24.1%)	112 (28.2%)	246 (22.0%)	
Unknown	7 (8.9%)	41 (7.3%)	<5 (1.3%)	23 (5.8%)	72 (6.4%)	
BMI (kg/m^2^)						0.084
Underweight (<18.5)	3 (4.1 %)	19 (3.6 %)	0 (0.0 %)	19 (5.1 %)	41 (3.7%)	
Normal/overweight (18.5–30)	61 (83.6 %)	394 (73.8 %)	57 (77.0 %)	258 (69.4 %)	770 (68.8%)	
Obese (>30)	9 (12.3 %)	121 (22.7 %)	17 (23.0 %)	95 (25.5 %)	222 (19.8%)	
Cardiovascular diseases						0.062
Yes	22 (27.8%)	157 (27.8%)	29 (36.7%)	139 (35.1%)	347 (31.0%)	
History non-tuberculous mycobacterial infection						0.098
Yes	<5 (5.1%)	43 (7.6%)	6 (7.6%)	46 (11.6%)	99 (8.8%)	
History pneumonia						0.010
Yes	27 (34.2%)	201 (35.6%)	33 (41.8%)	182 (45.8%)	443 (39.6%)	
Modified Medical Research Council Dyspnoea Score						**<0.001**
0	50 (63.3%)	220 (39.0%)	25 (31.6%)	90 (22.7%)	385 (34.4%)	
1	17 (21.5%)	204 (36.2%)	23 (29.1%)	117 (29.5%)	361 (32.3%)	
2	8 (10.1%)	85 (15.1%)	20 (25.3%)	95 (23.9%)	208 (18.6%)	
3	<5 (5.1%)	38 (6.7%)	10 (12.7%)	60 (15.1%)	112 (10.0%)	
4	0 (0.0%)	17 (3.0%)	<5 (1.3%)	35 (8.8%)	53 (4.7%)	
Smoking status						0.999
Current	<5 (2.5%)	17 (3.0%)	<5 (3.8%)	12 (3.0%)	34 (3.0%)	
Ex	30 (38.0%)	222 (39.4%)	30 (38.0%)	157 (39.5%)	439 (39.2%)	
Never	47 (59.5%)	325 (57.6%)	46 (58.2%)	228 (57.4%)	646 (57.7%)	
FEV1 %Predicted						**<0.001**
Mean (SD) (range)	90.171 (26.228) (22.510–169.570)	80.900 (24.421) (1.680–149.330)	75.252 (25.819) (30.310–138.410)	67.941 (26.313) (15.200–141.870)	76.624 (26.231) (1.680–169.570)	
FVC %Predicted						**<0.001**
N-Miss	10	58	7	51	126	
Mean (SD) (range)	106.800 (22.517) (61.280–174.240)	96.887 (22.813) (2.190–206.110)	90.711 (22.159) (47.290–142.300)	85.724 (23.669) (23.440–159.940)	93.238 (23.862) (2.190–206.110)	
Usual daily sputum volume (mL/day)						**0.007**
Mean (SD) (range)	26.149 (47.269) (2.960–250.000)	38.200 (61.827) (0.100–500.000)	47.735 (71.449) (1.480–250.000)	52.562 (64.906) (0.200–300.000)	43.775 (63.485) (0.100–500.000)	

Notes: Own construction, based on the BronchUK dataset. These figures refer only to the baseline information of the patients. Bold values indicate statistical significance at the p<0.05 level.

BMI, body mass index; BronchUK, Bronchiectasis Observational Cohort and Biobank UK; ED, emergency department; FEV, forced expiratory volume in 1 s; FVC, forced vital capacity.

The ethnic mix differed significantly across groups, with the more severe having mostly Caucasian background (table 1). The proportion with a documented history of pneumonia rose from 34% for the group with zero exacerbations to 46% for those requiring in-patient hospitalisation after an exacerbation. Recurrent or severe lower respiratory tract infections are linked to higher hospitalisation. The Modified MRC Dyspnoea score worsened with increasing severity: the percentage of patients with dyspnoea grade ≥3 increases from 5% (no exacerbations) to 24% (hospitalised).

Table 2 summarises the distribution of the number of exacerbations in the previous 12 months at baseline and at each BronchUK follow-up visit stratified by level of care. Exacerbation episodes that required only primary care intervention are labelled in table 2 as ‘Exacerbations with no secondary visits’; those that required emergency visit only without secondary (hospitalisation) care are labelled as ‘Exacerbations with ED but no secondary visits’; and those requiring hospitalisation are labelled as ‘Exacerbations with hospitalisation’. Table 2 records these visits for each BronchUK follow-up in the columns, with the rows 0, 1, 2, 2+ indicating the number of episodes per patient for baseline and subsequent BronchUK follow-ups (0, 1, 2 or ≥2). Categories are mutually exclusive at the episode level.

**Table 2:** Baseline exacerbations history and at follow-up

Variable	Baseline (N=1119)	Follow-up 1 (N=1007)	Follow-up 2 (N=795)	Follow-up 3 (N=605)	Follow-up 4 (N=411)	Follow-up 5 (N=252)
Exacerbations with no secondary visits						
0	287 (25.6%)	347 (34.5%)	282 (35.5%)	233 (38.5%)	136 (33.1%)	80 (31.7%)
1	209 (18.7%)	224 (22.2%)	171 (21.5%)	135 (22.3%)	100 (24.3%)	55 (21.8%)
2	172 (15.4%)	177 (17.6%)	147 (18.5%)	100 (16.5%)	80 (19.5%)	56 (22.2%)
3+	451 (40.3%)	259 (25.7%)	195 (24.5%)	137 (22.6%)	95 (23.1%)	61 (24.2%)
Exacerbations with ED but no secondary visits						
0	968 (86.5%)	947 (94.0%)	754 (94.8%)	571 (94.4%)	398 (96.8%)	240 (95.2%)
1	102 (9.1%)	42 (4.2%)	29 (3.6%)	31 (5.1%)	12 (2.9%)	9 (3.6%)
2	29 (2.6%)	11 (1.1%)	10 (1.3%)	3 (0.5%)	1 (0.2%)	3 (1.2%)
2+	20 (1.8%)	7 (0.7%)	2 (0.3%)	0 (0.0%)	0 (0.0%)	0 (0.0%)
Exacerbations with hospitalisation						
0	892 (79.7%)	860 (85.4%)	673 (84.7%)	523 (86.4%)	359 (87.3%)	202 (80.2%)
1	137 (12.2%)	99 (9.8%)	74 (9.3%)	63 (10.4%)	37 (9.0%)	34 (13.5%)
2	49 (4.4%)	26 (2.6%)	31 (3.9%)	11 (1.8%)	11 (2.7%)	11 (4.4%)
2+	41 (3.7%)	22 (2.2%)	17 (2.1%)	8 (1.3%)	2 (1.0%)	5 (2.0%)

Notes: Own construction, based on the BronchUK dataset.

BronchUK, Bronchiectasis Observational Cohort and Biobank UK; ED, emergency department.

For events managed without secondary care, the proportion reporting no exacerbation increased from 25.6% at baseline to 34%–39% over follow-ups, while ≥2 events declined from 40.3% to 22%; the one and two event categories remained stable (19%–24% and 16%–22%, respectively). For ED exacerbations, zero increased from 86.5% to 94%–97%, one fell from 9.1% to 3%–5%; two were uncommon (1%–3%) and ≥2 became negligible by later visits. For hospitalised exacerbations, no admission rose from 79.7% at baseline to 85%–87% through most follow-ups, with declines in ≥2 admissions (from 3.7% to 1%–2%) and generally lower proportions with one admission (9%–14%).

**Figure 1:**
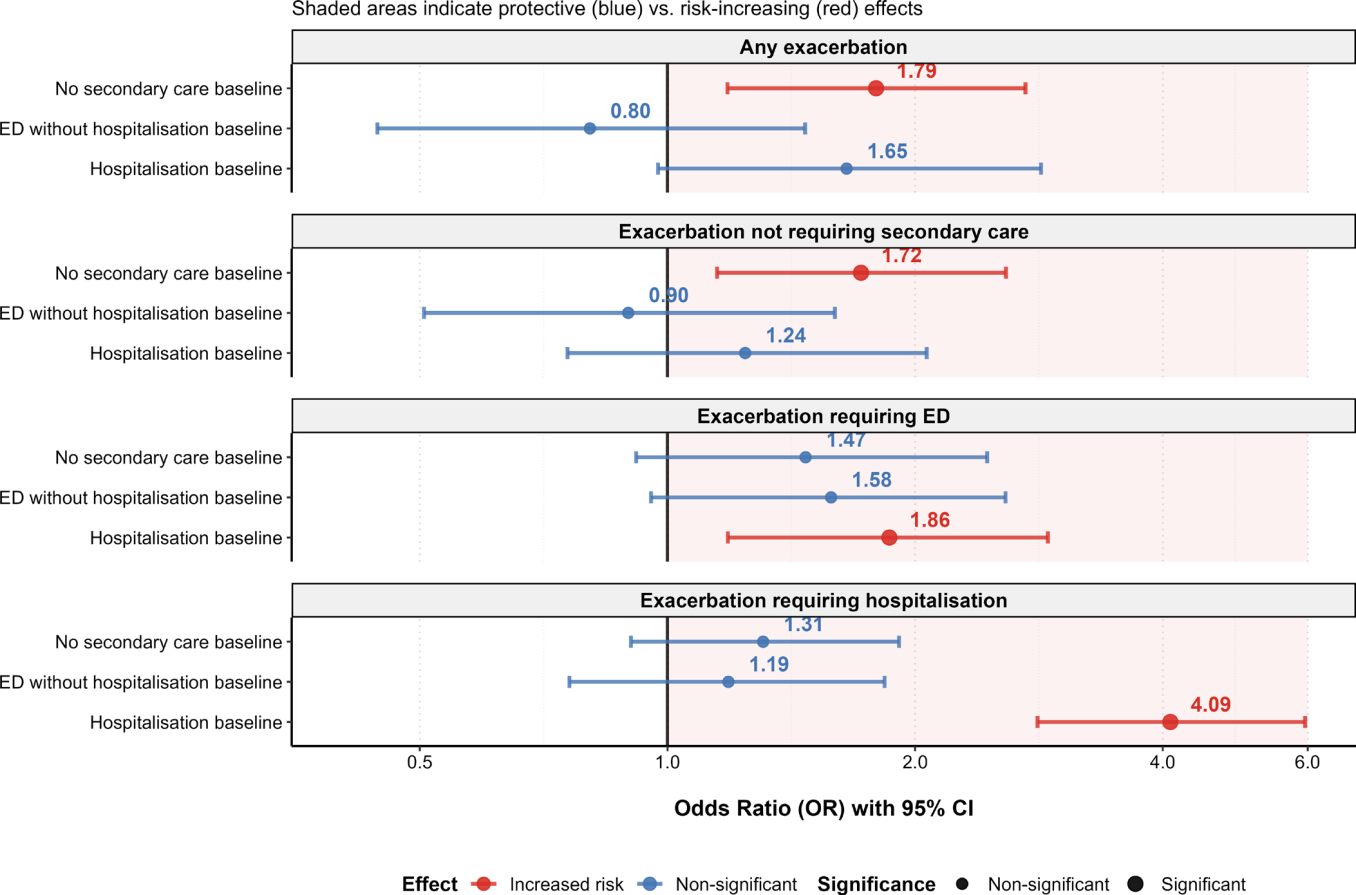
ORs for follow-up exacerbations by baseline status. Error bars represent 95% CIs. Reference line at OR=1.0. ED, emergency department.

Figure 1 reports the ORs from our regression analysis of baseline exacerbations on exacerbations during the follow-up period for individuals having any exacerbation and stratified by whether those suffering from an exacerbation at baseline required primary care only over the whole follow-up period; ED care only over the whole follow-up period; and those requiring hospitalisation over the whole follow-up period. The results demonstrate that experiencing an exacerbation not requiring secondary care at baseline doubles the odds of having any future exacerbation, as well as an exacerbation not requiring secondary care. Moreover, experiencing an exacerbation requiring hospitalisation at baseline doubles the odds of a future exacerbation requiring ED care and triples the odds of requiring inpatient care.

In estimating the costs of treatment, in-patient hospitalisation costs for BE patients are split into elective admissions with a range of treatment costs between £797 and £3193 per hospital episode the costs reflecting CC scores over a range 0–5, and emergency conditions where the in-patient treatment costs range from £1791 to £5152 again reflecting a range of CC scores. UK accident and emergency room (A&E) tariffs also reflect different levels of severity, ranging from £147 to £455 per visit. We applied conservative lower-bound unit costs (table 3): £1791 (emergency inpatient), £239 (ED) and £48 (primary care).

**Table 3:** Unit costs

Healthcare settings	Unit costs
Primary care visit	£48
ED care	£239 (£147–£455)
In-patient care	£1791 (£797–£3193)
CT scan	£113
Follow-up out-patient visit	£107
One prescription cost per visit	£28

Note: Own construction, prices being 2024/2025 National Health Service prices.

ED, emergency department.

The pattern of treatment costs draws on the utilisation data as reported in table 2. The treatment costs are reported annually for the baseline and 5 years of follow-up. It is common practice to report net present value figures through applying standard discount rates (the National Institute for Health and Social Care (NICE) in the UK uses a discount factor value of 1/ (1+0.035)^N^, where N is the number of years for present value to be returned and 0.035 is their discount factor), and we duly present undiscounted and discounted estimates of treatment costs over the 5-year period. Table 3 reports the highest level of costs at baseline are associated with inpatient treatments (£641 178), the next highest being primary care visits as the utilisation is highest (£91 002) and ED (A&E) costs are estimated at £52 580, giving total baseline costs of £784 760. Over the full 5-year follow-up period, the total inpatient treatment costs are £1 792 791 (discounted at 3.5%, these are £1 704 558), the next highest being primary care visits £284 596 (discounted, these are £269 179), followed by ED (A&E) treatment costs of £97 034 (£94 133 discounted). Table 4 reports the total treatment costs for this sample of 1119 BE patients at risk of suffering exacerbation, which is £2 174 003 (discounted £2 067 870). The average estimated cost per patient was £1943 for the full period. This corresponds to a weighted annual average of £495 per patient (including patients with no exacerbations). Specifically, the average cost was £2980 for individuals with one or more exacerbations, £4865 for those with two or more exacerbations, and £5875 for those with three or more exacerbations.

**Table 4:** Exacerbations costs across baseline and follow-ups

Total over baseline and follow-up undiscounted								Total over baseline and follow-up discounted							
	Baseline	Follow-up 1	Follow-up 2	Follow-up 3	Follow-up 4	Follow-up 5	Total		Baseline	Follow-up 1	Follow-up 2	Follow-up 3	Follow-up 4	Follow-up 5	Total
								Discount	1	0.966	0.934	0.902	0.871	0.842	
	GP visits only (unit cost=£48)							GP visits only (unit cost=£48)							
0	0	0	0	0	0	0	**0**	0	0	0	0	0	0	0	**0**
1	£9 979	£10 695	£8 164	£6 446	£4 775	£2 626	**£42 684**	1	£9 979	£10 333	£7 622	£5 814	£4 161	£2 211	**£40 119**
2	£16 424	£16 902	£14 037	£9 549	£7 639	£5 347	**£69 899**	2	£16 424	£16 330	£13 104	£8 613	£6 657	£4 502	**£65 630**
2+	£64 599	£37 098	£27 931	£19 623	£13 607	£8 737	**£171 596**	2+	£64 599	£35 843	£6 074	£7 699	£11 858	£7 357	**£163 430**
	Total GP undiscounted						**£284 178**	Total GP discounted							**£269 179**
	Outpatient visits only (unit cost=£239)							Outpatient visits only (unit cost=£239)							
0	0	0	0	0	0	0	**0**	0	0	0	0	0	0	0	**0**
1	£24 378	£10 038	£6 931	£7 409	£2 868	£2 151	**£53 775**	1	£24 378	£9 699	£6 470	£6 682	£2 499	£1 811	**£51 540**
2	£13 862	£5 258	£4 780	0	0	0	**£23 900**	2	£13 862	£5 080	£4 462	0	0	0	**£23 404**
2+	£14 340	£5 019	0	0	0	0	**£19 359**	2+	£14 340	£4 849	0	0	0	0	**£19 189**
	Total ED undiscounted						**£97 034**	Total ED discounted							**£94 133**
	Inpatient visits only (unit cost=£1791)							Inpatient visits only (unit cost=£1791)							
0	0	0	0	0	0	0	**0**	0	0	0	0	0	0	0	**0**
1	£245 367	£177 309	£132 534	£112 833	£66 267	£60 894	**£795 204**	1	£245 367	£171 313	£123 722	£101 769	£57 748	£51 271	**£751 190**
2	£175 518	£93 132	£111 042	£39 402	£39 402	£39 402	**£497 898**	2	£175 518	£89 983	£103 659	£35 538	£34 337	£33 175	**£472 210**
2+	£220 293	£118 206	£91 341	£42 984	0	£26 865	**£499 689**	2+	£220 293	£14 209	£85 268	£38 769	0	£22 620	**£481 158**
	**Total inpatient undiscounted**						**£1 792 791**	**Total inpatient discounted**							**£1 704 558**
	**Overall total undiscounted**						**£2 174 003**	**Overall total discounted**							**£2 067 870**

Note: All 2024/2025 National Health Service prices.

ED, emergency department; GP, general practitioner.

As a sensitivity analysis and given that these treatment costs are deliberately conservative estimates given our assumptions, we augmented the treatment costs through application of the current BTS guidelines.^13^ Noting that preparation tests will have been included in the treatment costs given above and drawing on BTS guidelines for BE,^13^ we assume that ideally individuals who are referred to secondary care will also incur CT scan costs (£113) as well as at least one follow-up outpatient visit cost (£107) and at least one prescription cost per visit (£28). We also increase the inpatient hospital cost for those with two or more exacerbations to that of a non-elective cost of £3173. Applying these additional costs to a sensitivity analysis increases total primary care costs to £479 945 (discounted £454 613), ED (A&E) costs to £260 126 (discounted £244 589) and inpatient care costs to £2 428 291 (discounted £2 313 479). Total treatment costs for the 1119 BE patients at risk of an exacerbation are then £3 160 362 (discounted £3 012 681) over the 5-year study follow-up period, mainly driven by inpatient costs (80%) within the sensitivity analysis.

Returning to our main results the highest annual estimates of treatment cost are associated with the baseline treatments, as this reflects the highest level of the study population, and is estimated to be £784 760, composed of £91 002 for primary care, £52 580 for those treated in A&E and £641 178 for those treated as inpatients. If we take 5 per 1000 as an approximation for BE population prevalence in the UK, with the UK population standing at 67.7 million this gives an estimated 338 500 cases (4). Given that our baseline (conservative) treatment cost is £784 760 for just over 1000 study individuals, that gives a baseline treatment cost of £784.76 per individual and a total UK cost of illness estimate of £265.6 million for 2024/2025.

## Discussion

This study, based on the BRONCK-UK population, is the first to report on a UK BE cohort at risk of exacerbation in terms of healthcare utilisation and costs using a unique dataset with a long-term follow-up period. Our findings align with international data indicating that exacerbations are the primary driver of healthcare costs in BE. A recent systematic review (7) highlighted significant cost variability across Germany, Spain and the USA, yet consistently identified exacerbation frequency and severity as key cost determinants. The most common BE aetiology was idiopathic or postinfectious. Longitudinally, baseline exacerbation history strongly predicted future events even after adjustment. Specifically, having one or more exacerbations that did not require secondary care at baseline was associated with roughly double the odds of recurrence in the follow-up period, compared with patients with no baseline exacerbation. Furthermore, a baseline exacerbation severe enough to require hospital admission markedly increased the likelihood of future ED visits and inpatient admissions, compared with those without a baseline exacerbation. These findings reinforce that exacerbations tend to beget more exacerbations, creating a cycle of healthcare utilisation.

Tracking the population as a series of cross-sections over a 5-year follow-up, we estimate the total treatment costs to be in the range £2 174 003 (discounted £2 067 870) to £3 160 362 (discounted £3 012 681). The average estimated cost per patient was £1943 (taking the lowest estimate) for the full period. This corresponds to a weighted annual average of £495 per patient (including patients with no exacerbations). Hospitalisation accounts for nearly 80% of the total spend on exacerbations. When disaggregated by the number of exacerbations, the average cost was £2980 for individuals with one or more exacerbations, £4865 for those with two or more, and £5875 for those with three or more. National data from England, Wales and Northern Ireland in 2013 showed a crude hospital admission rate for BE of about 88.4 per 100 000 population (95% CI 74.0 to 105.6) (7). The higher per-patient costs and admission frequency observed in our cohort likely reflect the fact that we focused on patients prone to exacerbations—a higher-risk (and thus higher-cost) subset of the overall BE population. Additionally, the relatively low number of ED-only visits reported (table 2) reflects UK clinical practice, where patients presenting to the ED with exacerbations are typically admitted, thereby shifting them into the inpatient category rather than being recorded as isolated ED events. Moreover, the cohort was recruited from centres often providing direct access to specialist review or admission, potentially bypassing routine emergency presentations.

BE remains underdiagnosed in clinical practice.^14^ It has been suggested that up to a quarter of individuals diagnosed with COPD have BE, particularly so in the more COPD severity groups.^15^ Similarly, Martínez-García *et al*^16^ reported that BE was present in 50% of patients with moderate-to-severe COPD, where it was independently associated with increased exacerbation frequency and mortality. As awareness increases and access to diagnostic CT scanning improves, the recorded prevalence of BE is expected to rise, especially in an ageing population. Our cost estimates will likely increase in tandem with better recognition of the disease. Using a prevalence of ~5 per 1000 in the UK and the current UK population (~67.7 million), this gives an estimated 338 000 BE cases nationwide (4). Given that we estimated a weighted annual average of £495 per patient (for just over 1000 study individuals), the total UK cost of illness estimate of £199 million for 2024/2025.

BE has previously had UK national audits according to prevailing BTS clinical care guidelines.^15 17^ Data suggest that access to and treatment with guideline-recommended therapies is not widespread.^15 17^ There are notably few cost-effectiveness studies in the prevailing guidelines.^18^ Our study adds UK-specific evidence on healthcare utilisation and costs, showing that exacerbations, both those managed outside hospital and those requiring admission, are key drivers of future service use. This directly complements the recent systematic review, which highlighted heterogeneous and largely non-UK evidence.^19^

Another important consideration is the current treatment landscape in BE. Data from large studies have shown significant use of inhaled corticosteroids in patients where there is no clear comorbidity suggesting these should be used (eg, asthma or COPD).^20^ Currently applied therapies are often empirical and are being actively studied to better determine their efficacy and cost-effectiveness, for example, mucolytics^21^ and bronchodilators.^22^ As emerging new treatments are developed in BE,^23 24^ these will need to be considered within future pathways in both clinical and cost-effectiveness.

The study is not without limitations. We had variable follow-up duration among patients, which poses analytical challenges. Although rate-based or time-to-event (survival) models can handle unequal follow-up, the degree of imbalance in our dataset was considerable, limiting the feasibility of those approaches. Applying Cox survival or negative binomial models in this context would have reduced statistical power and introduced additional assumptions that are hard to verify, particularly around the precise timing of the exacerbation which was not collected within the dataset. Therefore, we chose to use a baseline-anchored logistic regression model. This approach examines whether a patient’s exacerbation history and severity at baseline predict the occurrence of any subsequent exacerbation (yes/no outcome) during follow-up. By adjusting for the number of follow-up years observed for each patient, this method helps mitigate bias from differential follow-up (retention bias). While this sacrifices some granularity (eg, exact timing or count of exacerbations), it provides a robust analysis of the predictive value of baseline exacerbation status without overfitting sparse follow-up data. A further limitation concerns attrition over the 5-year follow-up: participation declined from 1119 patients at baseline to 252 at year 5. Patients who did not complete all follow-up visits may differ systematically from those who were retained—for example, if patients with more severe disease were more likely to be discharged, transferred or to die. Because the primary outcomes are defined on observed follow-up data (not requiring complete 5-year observation), and because regression models include the number of observed visits as a covariate, the impact of differential attrition on our main estimates is likely to be limited. Nevertheless, we cannot formally exclude informative censoring, and the assumption of missing at random for exacerbation outcomes, while reasonable, cannot be verified with the available data.

Future studies with more balanced follow-up or alternative designs (such as prospective registries) will be valuable to confirm our findings and explore time-to-event outcomes in more detail.

In conclusion, BE is increasingly recognised and its prevalence is expected to rise with an ageing population and greater access to CT scanning. We have demonstrated that the majority of costs incurred relate to exacerbations. These findings reinforce that exacerbations tend to beget more exacerbations, creating a cycle of healthcare utilisation. Improving access to effective therapies—and developing new ones—that can reduce exacerbation frequency is therefore of paramount importance. Such interventions would not only improve patients’ quality of life and outcomes, but also curb the high healthcare costs associated with recurrent exacerbations and associated hospital admissions.

## Supplementary material

10.1136/bmjresp-2026-004203 online supplemental file 1

## Data Availability

Data may be obtained from a third party and are not publicly available.
